# Biomarker Tools to Design Clinical Vaccines Determined from a Study of Annual Listeriosis Incidence in Northern Spain

**DOI:** 10.3389/fimmu.2016.00541

**Published:** 2016-11-29

**Authors:** Ricardo Calderon-Gonzalez, Hector Teran-Navarro, José María Marimon, Claudia González-Rico, Jorge Calvo-Montes, Elisabet Frande-Cabanes, Miriam Alkorta-Gurrutxaga, M. C. Fariñas, Luis Martínez-Martínez, Emilio Perez-Trallero, Carmen Alvarez-Dominguez

**Affiliations:** ^1^Grupo de Nanovacunas y vacunas celulares basadas en Listeria y sus aplicaciones en biomedicine, Instituto de Investigación Marqués de Valdecilla (IDIVAL), Santander, Spain; ^2^Servicio de Microbiología, Instituto de Investigación Sanitaria Biodonostia, Hospital Universitario Donostia, San Sebastián, Spain; ^3^CIBER de Enfermedades Respiratorias (CIBERES), Madrid, Spain; ^4^Sección de Enfermedades Infecciosas, Hospital Universitario Marques de Valdecilla, Santander, Spain; ^5^Servicio de Microbiología, Hospital Universitario Marques de Valdecilla, Santander, Spain; ^6^Departamento de Medicina y Psiquiatría, Universidad de Cantabria, Santander, Spain; ^7^Departamento de Biología Molecular, Universidad de Cantabria, Santander, Spain

**Keywords:** listeriosis, *Listeria*, zoonoses

## Abstract

Two regions of northern Spain, Gipuzkoa, and Cantabria present high annual incidence of listeriosis (1.86 and 1.71 cases per 100,000 inhabitants, respectively). We report that the high annual incidences are a consequence of infection with highly virulent *Listeria monocytogenes* isolates linked to fatal outcomes in elderly patients with cancer. In addition, listeriosis patients with cancer present low IL-17A/IL-6 ratios and significantly reduced levels of anti-GAPDH_1–22_ antibodies, identified as two novel biomarkers of poor prognosis. Analysis of these biomarkers may aid in reducing the incidence of listeriosis. Moreover, GAPDH_1–22_-activated monocyte-derived dendritic cells of listeriosis patients with cancer seem useful tools to prepare clinical vaccines as they produce mainly Th1 cytokines.

## Background

In Spain, increased cases of listeriosis were recorded between 2008 and 2014, with up to 1.15 cases per 100,000 inhabitants according to the last European summary report in 2014, signifying one of the highest incidences of listeriosis in Europe (1–4). Over this period, outbreaks were additionally documented in Austria, Portugal, Spain, and Denmark (1, 5–8). Listeriosis has only recently been listed as a notifiable disease in Spain since March 2015 (9), and therefore, the annual incidence may be even higher (1–5, 10, 11). Reduced immunocompetency of listeriosis patients was recently reported as a major risk factor for fatal outcomes (12). Epidemiologically specific *Listeria monocytogenes* genotypes show greater virulence, contributing to more human listeriosis cases (13, 14). Despite accumulating epidemiological data on listeriosis (1–14), effective immunological biomarkers are yet to be established that *a priori* appear to represent valuable tools to prepare clinical vaccines for patients at high risk of listeriosis, which may help to reduce the incidence of infectious disease.

The main purpose of this study was to establish the annual incidence of listeriosis in two northern Spain communities with voluntary reports of high numbers of listeriosis cases. We further explored potential immunological and epidemiological biomarkers that may serve as major risk factors of fatal outcomes as well as tools to generate clinical vaccines for patients at high risk of listeriosis.

### Study Description

Two northern Spain regions, Gipuzkoa in the Basque country (~700,000 inhabitants) and Cantabria (~350,000 inhabitants), were selected to calculate annual incidence, since these communities have voluntarily reported a high number of listeriosis cases since 2008 (1, 4, 5, 10, 11). We compiled data on genetic epidemiology, clinical manifestations, virulence of clinical isolates, and immunological parameters in listeriosis patients over the period of a year (August 2014–September 2015). This time period was selected to ensure that at least 5 months had passed after the January 2013–February 2014 outbreak (5), thus avoiding inclusion of any listeriosis cases from this epidemic in our annual study of incidence. Non-maternal/neonatal patients who met the laboratory criteria or mothers with laboratory-confirmed listeriosis infection in the fetus, stillborn, or newborn, according to the Commission Decision of 28/IV/2008, were classified as listeriosis cases (15). In total, nine human listeriosis episodes were detected in Gipuzkoa and six in Cantabria, being the annual incidences very high, 1.86 and 1.71 cases per 100.000 inhabitants, respectively. Bacteria were recovered from blood in all except three cases, where recovery was from ascitic fluid, stool, or urine. Seven cases (46.7%) were adults 45–65 years of age, and six (40%) involved the elderly (>70 years). Seven patients presented solid- or blood-related tumors under chemotherapeutic treatment (50%), including two deceased cases, lung adenocarcinoma, squamous cell glottis carcinoma, lung and bladder carcinoma, glioblastoma multiforme, hepatocarcinoma, and multiple myeloma IgGλ. Two episodes (13.3%) corresponded to autoimmune diseases, glomerulonephritis, and cutaneous lupus, two occurred during pregnancy, including the child of a patient infected with meningitis (13.3%), two episodes of meningitis or bacteremia were associated with elderly patients (13.3%), one case related to a combined liver*–*kidney transplantation (6.6%), and one case of sporadic listeriosis occurred in an apparent non-risk patient (6.6%) (Table 1, 2014–2015).

**Table 1:** **Listeriosis patients: clinical manifestations and treatments**.

Patients code^a^-age (y), period 2014–2015	Clinical manifestations	Type of infection	Listeriosis treatment	Other treatments
HUD005-74 (*deceased*)	Lung adenocarcinoma	Bacteremia	Ampicillin	Cisplatin + holocraneal radiotherapy
HUD006-60	Multiple myeloma IgGλ	Bacteremia, meningitis	Ampicillin + gentamicin	Melphalan + radiotherapy + lenalodomide, velcade and dexamethasone
HUD007-30	Pregnancy, cesarean	Corioamnionitis	NT	NT
HUD008-0	Premature neonate	Meningitis	Ampicillin	NT
HUD009-74	None	Meningitis	Ampicillin	Lisionopril
HUD010-90	Rhabdomyolysis	Bacteremia	Ampicillin	NT
HUD011-54	None	Bacteremia, blood diarrheal	Ampicillin	NT
HUD012-65	Type I hepatorenal syndrome	Peritonitis	Ampicillin	NT
HUD013-59	Hepatocarcinome	Bacteriuria	Ampicillin	Surgery
HUMV006-76 (*deceased*)	Prostate adenocarcinoma	Bacteremia	ND	Taxocel
HUMV007-51	Squamous cell glottis carcinoma	Bacteremia	Amoxicillin + clavulanic + ampicillin	Radiotherapy + cetuximab
HUMV009-54	Lung and bladder carcinoma	Bacteremia	Levofloxacin	Cisplatin-etoposide
HUMV010-57	Glomerulonephritis	Sepsis	Meropenem + ampicillin	Mycophenolate mofetil + everolimus
HUMV012-84	Cutaneous lupus	Bacteremia	Amoxicillin + clavulanic + ampicillin	Prednisone
HUMV013-71	Glioblastoma multiforme	Bacteremia	Augmentin	Temozolamide + radiotherapy

**Patients code-age (y) selection in 2012–2014^b^**	**Clinical manifestations**	**Type of infection**	**Listeriosis treatment**	**Other treatments**

HUD001-57	Squamous cell glottis carcinoma	Bacteremia	Ampicillin	Cisplatin
HUD002-36	Splenectomized autoimmune-hypertiroidism	Bacteremia	Ampicillin	Tirodril
HUD003-30	Pregnancy	Bacteremia	NT	NT
HUD004-32	Pregnancy, cesarean-2nd twin lost	Bacteremia	NT	NT
HUMV001-89	Arteritis of giant cells	Acute meningoencephalitis	Ampicillin	Prednisone
HUMV002-65	Hepatocellular carcinoma	Bacteremia	Ampicillin	Ablation by microwaves
HUMV003-60	Guillain–Barre syndrome, sarcoidosis	Bacteremia, hepatic abscess	Ampicillin + gentamicin	Prednisone
HUMV004-56	Cirrhosis-hepatic transplant	Brain abscess	Ampicillin	Prednisone + mycophenolate mofetil + tacrolimus
HUMV005-49	Cutaneous primary lymphoma of giant cells	Bacteremia	Ampicillin + gentamicin	Rituximab + local radiotherapy

*^a^Clinical manifestations and treatments of listeriosis patients during August 2014–September 2015 or in a ^b^selection of patients in 2012–2014. Patients identified by internal codes. HUD, Hospital Universitario de Donostia (Gipuzcoa); HUMV, Hospital Universitario Marqués de Valdecilla (Santander, Cantabria). Age of the patient in years (y). NT, no treatment*.

### Microbiological Characterization of Invasive *L. monocytogenes*

We collected invasive *L. monocytogenes* from 15 clinical isolates and serotyped the strains by agglutination (Listeria-O-antisera, Difco) and performed multilocus sequence typing (MLST) to establish *L. monocytogenes* sequence types (STs) using the primers and conditions described on the Pasteur Institute web page (http://bigsdb.pasteur.fr/listeria/listeria.html) (16–18). In total, 74% (11/15) invasive *L. monocytogenes* were classified as serotype 4b, 7% (1/15) as serotype 1/2b, and 23% (3/15) as serotype 1/2a, with 12 and 3 clinical isolates of lineages I and II, respectively. MLST genotyping revealed the following distribution: 43% ST1, 14% ST219, and 7% each of ST6, ST26, ST37, ST87, ST213, and ST391 genotypes. These distributions differ from those registered on Pasteur Institute database (column^e^ in Table 2), something attributable to the limited number of samples. The ST87 ST of the Gipuzkoa outbreak in 2014 (5) was detected in Cantabria as well as other regions in northern Spain (10). The most prevalent ST in our clinical isolates of invasive *L. monocytogenes* was ST1, consistent with previous reports (10, 13, 14, 17, 18), followed by ST219. With the aid of M. Lecuit and M. Maury (Institut Pasteur, Paris, France), we assigned the corresponding clonal complexes (CC) to these STs. The most prevalent CC were the hypervirulent clones CC1 (ST1) and CC4 (ST219) (column^e^ in Table 2). In agreement with data obtained from the detailed investigation performed in France (14), CC1 and CC4 hypervirulent clones corresponded to listeriosis cases of meningitis or materno-fetal transmission. We additionally detected another hypervirulent clone, ST6 (CC6), isolated from a patient with squamous cell glottis carcinoma (Tables 1 and 2, 2014–2015). The data collectively suggest that bacterial isolates of our listeriosis patients have a highly virulent phenotype.

**Table 2:** **Listeriosis patients: microbiological and epidemiological parameters**.

Patients code^a^-age (y)^c^, period 2014–2015	Serotype^d^	Lineage^d^	Sequence type (ST)^f^	HUD/HUMV distribution^e^ (%)	Pasteur distribution^e^ (%)
HUD005-74^b^ (*deceased*)	1/2a	II	37 (CC37)	7.69	0.53
HUD006-60^b^	4b	I	219 (CC4)	15.38	0.13
HUD007-30	4b	I	1 (CC1)	53.84	10.27
HUD008-0	4b	I	1 (CC1)	53.84	10.27
HUD009-74	4b	I	219 (CC4)	15.38	0.13
HUD010-90	4b	I	1 (CC1)	53.84	10.27
HUD011-54	4b	I	1 (CC1)	53.84	10.27
HUD012-65	4b	I	1 (CC1)	53.84	10.27
HUD013-59^b^	4b	I	1 (CC1)	53.84	10.27
HUMV006-76^b^ (*deceased*)	4b	I	213 (CC213)	9.09	0.03
HUMV007-51^b^	4b	I	6 (CC6)	9.09	1.69
HUMV009-54^b^	1/2b	I	87 (CC87)	9.09	0.69
HUMV010-57	1/2a	II	391 (CC89)	9.09	0.28
HUMV012-84	4b	I	1 (CC1)	18.18	10.27
HUMV013-71^b^	1/2a	II	26 (CC26)	9.09	0.38

**Patients code^a^-age (y)^c^, selection in 2012–2014^h^**	**Serotype^d^**	**Lineage^d^**	**Sequence type (ST)^f^**	**HUD/HUMV distribution (%)^e^**	**Pasteur distribution (%)^g^**

HUD001-57^b^	4b	I	4 (CC4)	NA	2.50
HUD002-36	1/2b	I	87 (CC87)	NA	0.69
HUD003-30	1/2b	I	87 (CC87)	NA	0.69
HUD004-32	4b	I	1 (CC1)	NA	10.27
HUMV001-89	4b	I	2 (CC2)	NA	6.67
HUMV002-65^b^	1/2b	I	3 (CC3)	NA	7.92
HUMV003-60	4b	I	54 (CC54)	NA	0.22
HUMV004-56	4b	I	666 (CC666)	NA	0.06
HUMV005-49^b^	4b	I	1 (CC1)	NA	10.27

*^a^List of listeriosis patients with tumors during August 2014–September 2015 and in the selection 2012–2014. Patients identified by internal codes as in Table 1*.

*^b^Patients with cancer*.

*^c^Age of patient in years (y)*.

*^d^Distribution of ST per institution, expressed as a percentage*.

*^e^Serotypes and lineages of clinical bacterial isolates measured via PCR multiplex*.

*^f^Sequence type, ST, of clinical bacterial isolates measured using MLST and complex clones in parentheses in the period 2014–2015 (14)*.

*^g^Distribution of ST obtained from Institut Pasteur website as of October 2016, expressed as a percentage (*n* = 3165)*.

*^h^Selection of listeriosis patients over January 2012–July 2014 based on the predominant clinical manifestations (see Table 1). NA, not applied*.

### *In Vitro* and *In Vivo* Replication of Clinical *L. monocytogenes* Isolates

**Figure 1:**
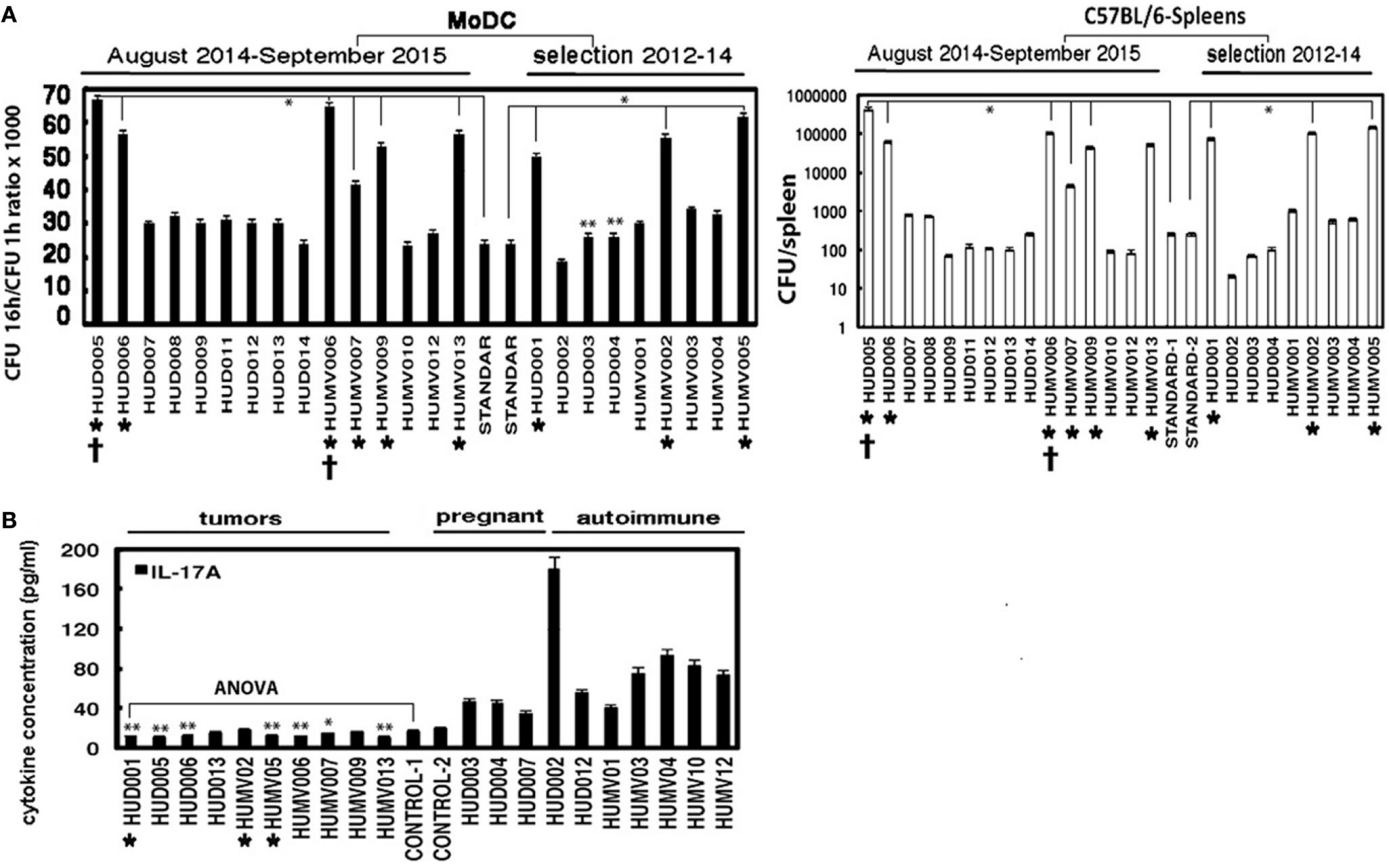
**Virulence of clinical isolates of listeriosis patients and immunological biomarkers**. **(A)** *Left panel* shows the replication index of *L. monocytogenes* isolates measured as RI in MoDC (black bars). Results are expressed as the mean ± SD of triplicates. *P ≤ 0.05; **P ≤ 0.01, *right panel* shows the CFU recovered in the spleens of mice. Results are expressed as the mean ± SD of triplicates. *P ≤ 0.05. Asterisks under the bars indicate 1000-fold higher replication indexes than standard *Listeria* strains. Standard-1 corresponds to 10403S strain and standard-2 to EGD strain. ^†^Symbols under the bars correspond to deceased patients. **(B)** IL-7A levels (pg/ml) in sera of all patients with listeriosis (2014–2015 and 2012–2014) clustered by three main clinical manifestations (tumors, pregnancy, and autoimmune disease) measured *via* flow cytometry (Methods in Supplementary Material). *P ≤ 0.05; **P ≤ 0.01. Controls are healthy donors. Results are expressed as the mean ± SD.

The *in vitro* replication of invasive *L. monocytogenes* isolates was assayed in human monocyte-derived dendritic cells (MoDC) (Methods in Supplementary Material) (19) infected at a MOI of 20:1 (bacteria:cells) and calculated as replication index (RI) as reported (20–22). This parameter is considered an indicator of bacterial growth in dendritic cells (DC) comparable to *in vivo* virulence in spleen after 72-h post-infection with *L. monocytogenes* (20, 21). Notably, *L. monocytogenes* isolates of patients with tumors presented a 1000-fold higher growth ratio in MoDC (asterisks and black bars in Figure 1A, 2014–2015) than two standardized *L. monocytogenes* controls (14, 17, 20, 23). The highest invasive *L. monocytogenes* virulence corresponded to that in deceased patients (†, Figure 1A, left plot). We additionally examined *in vivo* virulence using standard procedures to measure the number of viable bacteria (CFU) that reached the spleens in C57BL/6 mice, a mice model highly resistant to listeriosis (20, 21, 23). Inoculation of mice with bacterial isolates from listeriosis patients with tumors, including those of deceased patients, induced 1000-fold higher levels of viable bacteria (CFU) in spleen, relative to those inoculated with isolates from the remaining listeriosis patients or either of the two standards, suggestive of very high virulence (right plot, Figure 1A). The similar *in vitro* and *in vivo* data suggest that measurement of listeriosis virulence in MoDC could provide a valid, low-cost, and rapid method without the necessity of inoculating mice. MLST genotyping of these highly virulent clinical isolates of listeriosis patients with tumors under active chemotherapeutic treatment [ST37 (CC37), ST219 (CC4), ST213 (CC213), ST6 (CC6), ST87 (CC87), and ST26 (CC26)] revealed the presence of at least two hypervirulent complex clones previously reported in France, CC4 and CC6 (14), which, in our study, corresponded to those in tumor patients subjected to chemotherapeutic regimens of cisplatin-etoposide (CC4) and cetuximab (CC6) (Tables 1 and 2). The genotype of the 2014 outbreak strain in northern Spain, ST87 (CC87), was isolated from a listeriosis patient with tumors subjected to chemotherapy with cisplatin-etoposide, confirming our previous suggestion that CC87 is a hypervirulent complex clone (5). Two other strains of particular interest were ST37 (CC37) and ST213 (CC213), isolated these strains from deceased patients with tumors under chemotherapy treatment with cisplatin and taxocel. These strains displayed the highest virulence and emerged as novel hypervirulent strains associated with listeriosis patients containing tumors. We concluded that patients with tumors treated with chemotherapy present a high-risk factor for invasive *L. monocytogenes* infection, consistent with earlier reports (12), since this patient population is highly sensitive to previously reported hypervirulent strains, such as CC4, CC6 (17), or CC87 (5), and newly emerging hypervirulent complex clones, CC37 or CC213, that may cause fatal outcomes (this study).

### Listeriosis Biomarkers of Poor Prognosis

Since a major risk for invasive *L. monocytogenes* infection in our patients is reduced immunocompetency, it is possible that specific immunological parameters contribute more significantly as risk factors. Two parameters appear relevant for the immune status of invasive *L. monocytogenes*-infected individuals. First, the Th1/Th17-Th2 cytokine balance (24–27) is a determinant of bacterial dissemination success, especially in elderly populations with diminished Th17 responses (28). Second, the levels of antibodies against the *L. monocytogenes* GAPDH virulence factor epitope, GAPDH_1–22_, are relevant biomarkers. Anti-GAPDH_1–22_ antibody titers reflect the ability of GAPDH to confer listeriosis protection (20, 21, 29, 30). Blood tests of listeriosis patients reflected the expected parameters: low lymphocyte numbers (3%–6%), normal monocyte values (2%–13%) (Table 3, blood test columns), and high neutrophil counts in several patients (≥ 75%–94.9%), with immature granulocytes in certain patients (28.5%), and especially high numbers in the two deceased patients. Patients with tumors, in particular deceased patients, presented very low IL-17A/IL-6 ratios and high levels of IL-6 and IL-10 in sera, while IFN-γ levels were similar as controls (Table 3, 2014–2015; Figure 1B). However, listeriosis patients with autoimmune diseases or miscarriages showed high levels of IL-17A/IL-6 ratios (Table S1 in Supplementary Material). These results indicate that impairment of the Th17/Th2 balance toward Th2 responses may present a risk factor leading to fatal outcomes.

**Table 3:** **Blood parameters of listeriosis patients: patients with tumors**.

Patients code^a^-age^b^, period 2014–2015	Blood test^c^ (%)			Cytokines^d^ and antibodies^e^ in sera				
	PMN (42–75)	Lymph (20–51)	Mo (1–13)	IL-17A/IL-6	IFN-γ	IL-6	IL-10	Anti-GAPDH_1–22_
HUD005-74 (*died*)	92^#^	4.9	2.1	1.20 ± 0.1**	4.7 ± 0.1	9.4 ± 0.4	4.9 ± 0.2	0.02 ± 0.1
HUD006-60	87	5.8	6.6	1.20 ± 0.2**	4.5 ± 0.1	10.2 ± 0.1	5.1 ± 0.3	0.38 ± 0.2**
HUD013-59	82.5	6.0	11.1	1.10 ± 0.1	4.0 ± 0.1	10.9 ± 0.2	5.3 ± 0.2	0.31 ± 0.2**
HUMV006-76 (*died*)	55^#^	9.0	5.0	1.75 ± 0.1	4.4 ± 0.2	6.6 ± 0.2	4.1 ± 0.2	0.05 ± 0.1
HUMV007-51	84^#^	5.0	10.2	2.80 ± 0.1*	2.2 ± 0.1	5.1 ± 0.2	1.6 ± 0.1	0.5 ± 0.2*
HUMV009-54	95	3.0	1.1	2.80 ± 0.2	1.4 ± 0.2	6.0 ± 0.1	4.6 ± 0.1	0.7 ± 0.2**
HUMV013-59	89	5.2	4.7	1.20 ± 0.1**	0.8 ± 0.1	9.0 ± 0.5	3.8 ± 0.1	0.2 ± 0.1**
**CONTROL-52**	**45**	**43**	11	**4.83 ± 0.2**	**2.4 ± 0.2**	**3.1 ± 0.2**	**2.4 ± 0.1**	**0.16 ± 0.1**

**Patients code-age, selection in 2012–2014^f^**	**Blood test (%)**			**Cytokines and antibodies in sera**				
	**PMN (42–75)**	**Lymph (20–51)**	**Mo (1–13)**	**IL-17A/IL-6**	**IFN-γ**	**IL-6**	**IL-10**	**anti-GAPDH_1–22_**

HUD001-57	76^#^	14	8	3.25 ± 0.1**	4.6 ± 0.2	8.0 ± 0.4	4.0 ± 0.1	1.14 ± 0.1**
HUMV002-65	90	22	4	0.95 ± 0.1	2.1 ± 0.1	19 ± 0.9	2.4 ± 0.1	0.80 ± 0.2*
HUMV005-49	87^#^	5	8	1.59 ± 0.2**	2.6 ± 0.1	7.9 ± 0.3	4.0 ± 0.1	0.50 ± 0.1**
**CONTROL-51**	**42**	**46**	**12**	**4.83 ± 0.2**	**2.3 ± 0.2**	**3 ± 0.2**	**2.3 ± 0.1**	**0.15 ± 0.1**

*^a^Listeriosis patients with tumors from August 2014 to September 2015 (asterisks) and patients with other clinical manifestations (miscarriage, renal transplant, and autoimmune as controls of parameters in other patients). Patients were identified by internal codes as in Table 1*.

*^b^Age of patient in years*.

*^c^Blood tests correspond to routine assays indicating the percentages of neutrophils (PMN), lymphocytes (Lymph), and monocytes (MO). Controls are healthy donors. Values in parentheses refer to ranges of normal values*.

*^#^Patients with immature granulocytes*.

^d^Cytokine concentration in sera (pg/ml) and IL-17A/IL-6 ratios expressed as the mean of ratio units (U) of triplicates ± SD. **P ≤ 0.01; *P ≤ 0.05.

^e^Sera of listeriosis patients were examined for peptide-ELISA (anti-GAPDH_1–22_) (21, 30). Results are presented as optical units (OD) and mean values ± SD of triplicate experiments. **P ≤ 0.01; *P ≤ 0.05.

*^f^Selected listeriosis patients with malignancies in 2012–2014*.

High levels of IgG antibodies (OD ≥ 2.0) against the bacterial epitope, GAPDH_1–22_ (20, 21), were detected in listeriosis patients with miscarriages, autoimmune diseases, or kidney transplants (OD of 2.5–4.5 OD) (Table S1 in Supplementary Material). The majority of listeriosis patients containing tumors had low IgG anti-GAPDH_1–22_ levels (OD of 0.2–0.8), presenting the deceased patients basal levels (0.02–0.05 OD) (Table 3, anti-GAPDH_1–22_ column). Anti-listeriolysin O (LLO) antibodies were detected at extremely low levels in all listeriosis patients (≤0.15 OD), eliminating the possibility of using LLO antibodies as biomarkers (data not shown). Accordingly, we conclude that low levels of anti-GAPDH_1–22_ antibodies, together with low IL-17A levels, are two valid immunological biomarkers of poor prognosis in listeriosis patients with tumors under chemotherapeutic treatment.

Next, we selected representative listeriosis patients between 2012 and 2014 from our institutional biobanks containing stores of bacterial isolates and cells to establish the epidemiological and immunological parameters of interest. In total, 81 cases of listeriosis between 2012 and 2014 occurred, including 16 deaths, 9 with tumors, 2 autoimmune diseases, 1 cirrhosis, 2 fetuses (miscarriages), and 1 child (stillbirth). Our selection of listeriosis patients was based on those representative of the most prevalent clinical manifestations in our institutions, specifically, those with tumors, autoimmune disease, miscarriages, and renal or hepatic transplants (5, 4, 11). Consequently, we selected three listeriosis patients with tumors, squamous cell glottis carcinoma treated with cisplatin, hepatocellular carcinoma treated with microwave ablation, and cutaneous primary lymphoma of giant cells subjected to rituximab treatment; three listeriosis patients with autoimmune diseases, an autoimmune hyperthyroidism treated with tirodril, arteritis of giant cells, and a Guillain–Barre syndrome treated with prednisone, two listeriosis-associated miscarriages in pregnant women, and one listeriosis patient with cirrhosis and hepatic transplant treated with several immune-suppressors (prednisone, mycophenolate mofetil, and tacrolimus) (Table 1, selection in 2012–2014 rows). We confirmed the isolation of highly virulent clinical isolates as CC1, CC3, and CC4 from listeriosis patients with tumors subjected to chemotherapy. Verification of virulence *in vitro* using MoDC or *in vivo* revealed 1000-fold higher RI values and CFU than the standard bacteria (Table 2, selection in 2012–2014 rows; Figure 1A, asterisks under selection 2012–2014). Second, we detected in the listeriosis patients with cancer, low levels of IL-17A (bars with asterisk in Figure 1B; Table 3, selection in 2012–2014 rows) and anti-GAPDH_1–22_ antibody levels (anti-GAPDH_1–22_ column in Table 3, selection 2012–2014). All other listeriosis groups of patients (autoimmune, hepatic transplanted, or miscarriages) presented bacterial isolates with virulence similar to standard strains (Figure 1A, selection 2012–2014) as well as high IL-17A and IgG anti-GAPDH_1–22_ levels (Table 3; Table S1 in Supplementary Material, selection 2012–2014).

**Figure 2:**
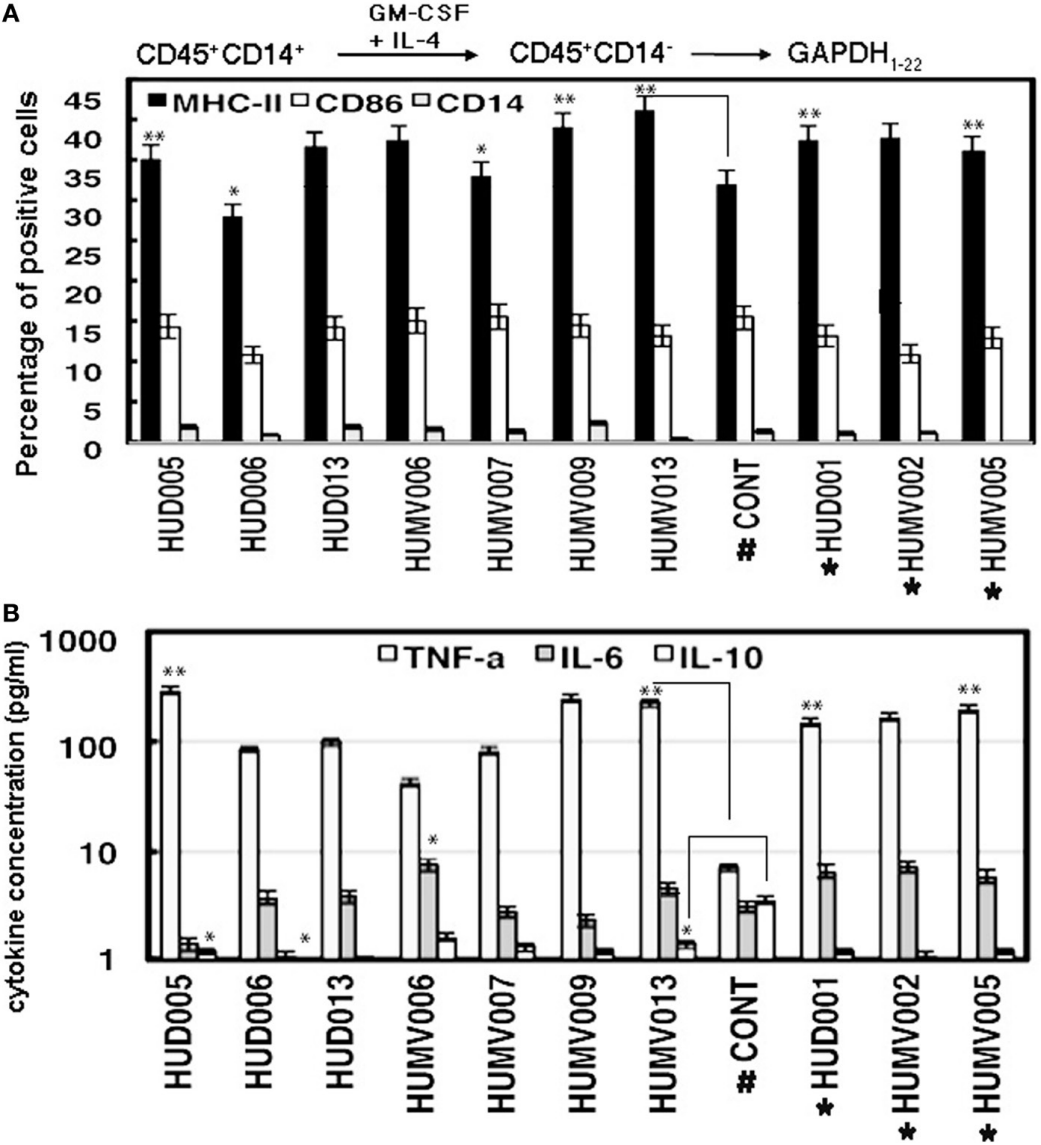
**Activation of MoDC from listeriosis patients with cancer**. **(A)** Differentiated and GAPDH_1–22_-activated MoDC from listeriosis patients with tumors (Methods in Supplementary Material) were examine for cell surface expression. Results are expressed as the percentages of positive cells ± SD. *P ≤ 0.05; **P ≤ 0.01. **(B)** Cytokine concentration in supernatants of GAPDH_1–22_-activated MoDC. Results are expressed as the mean of cytokine concentrations (pg/ml) ±SD and ANOVA was applied. *P ≤ 0.05; **P ≤ 0.01.

Since the main risk factors in our listeriosis patients with tumors subjected to chemotherapeutic treatments were identified as reduced Th17 immunocompetency, we hypothesized that this biomarker parameter could be improved in activated MoDC. We based our hypothesis in the ability of DC to elicit pro-inflammatory Th1/Th17 and effective B cell-CD4^+^ T cell immune responses (21, 26, 27), even in clinical trials for patients with cancer (31). For this purpose, we activated MoDC from all listeriosis patients with cancer vaccine with GAPDH_1–22_ peptide as described (20, 21) and observed in all MoDC an activated phenotype, CD45^+^MHC-II^+^CD86^+^CD14^−^, similar to activated MoDC from controls (19–21) (Figure 2A). We also observed that GAPDH_1–22_-activated MoDC from listeriosis patients with cancer released high levels of TNF-α while low levels of IL-6 and IL-10 (Figure 2B), suggesting a shifting toward Th1 pattern. Finally, we have not addressed here the type of food associated with our listeriosis patients but in all cases, listeriosis infection was related to consumption of contaminated food as reported in other epidemiological studies (1–14, 17).

## Discussion

Epidemiological studies of European outbreaks and case studies, coordinating clinical data and bacteriological genotyping, have led to significant progress in listeriosis surveillance and recently revealed the presence of hypervirulent strains in clinical isolates (5–14, 16–18) as well as reduced immunocompetency as a main risk factor (12). Our data on listeriosis cases collected from two northern Spain communities, Gipuzkoa and Cantabria, over the course of a year (obtained to calculate annual listeriosis incidences) were in agreement with these findings. Our study concludes that the most important risk factors for listeriosis are the reduced Th17 immune competency of patients with cancer associated with the chemotherapy received and their high sensitivity to infection with hypervirulent strains, along with low levels of two immunological biomarkers, IL-17A/IL-6 ratios, and IgG anti-GAPDH_1–22_ antibodies.

First, fatal outcomes were only observed in listeriosis patients with cancer receiving chemotherapy and not in autoimmune or renal-hepatic transplanted patients receiving immune-suppressants or in the elderly, arguing low Th17 immune abilities caused by chemotherapy were risk factors more important than immune-senescence or autoimmunity.

Second, we presented a simple and rapid method to examine virulence in resting MoDC that revealed a correlation of *L. monocytogenes* hypervirulent strains with mortality in listeriosis patients with tumors receiving chemotherapy. In particular, these patients display high sensitivity not only to infections with hypervirulent invasive *L. monocytogenes* strains associated with meningitis, such as CC1, CC4, or CC6 (14) and other hypervirulent bacterial strains prevalent in northern Spain communities, such as CC87 (5, 10), but also to newly reported hypervirulent invasive *L. monocytogenes* strains, such as CC37, CC26, and CC213, isolated from deceased patients (this study). Third, two biomarkers in the sera of patients with poor listeriosis prognosis were distinguished: (i) low IL-17A/IL-6 ratios that reflected a shift in the immune balance toward Th2 anti-inflammatory responses and (ii) low production of anti-GAPDH_1–22_ antibodies that suggested reduced CD4^+^ T cell–B cell responses. Dysfunction of Th17 immune responses in listeriosis patients with tumors under chemotherapeutic treatment appears to explain the high bacterial loads and reduced production of antibodies (24, 28), as IgG anti-GAPDH_1–22_ antibodies. Listeriosis patients with other co-morbidities presented better Th17 and CD4^+^ T cell immune responses. High levels of IL-6 were also reported in bacterial meningitis of children and experimental listeriosis (32, 33), suggesting this cytokine plays a relevant role. Moreover, IL-10 high levels were also reported to increase in listeriosis of aged mice with high bacterial loads (28). Therefore, high levels of Th2 cytokines (IL-6 or IL-10) seemed related with severe listeriosis.

Vaccinations are best measures to prevent opportunistic infections, such as invasive *L. monocytogenes*. In this regard, DC or synthetic vaccines containing GADPH_1–22_ epitopes and targeted to DC seemed to confer protection against experimental listeriosis, promoting Th1-Th17 and CD4^+^-CD8^+^ T cell immune responses (20, 21, 30, 34). Here, we report that GAPDH_1–22_ epitopes can activate MoDC of listeriosis patients with cancer receiving chemotherapy to release high levels TNF-α while low production of IL-6 and IL-10, a clear Th1 cytokine pattern. These findings should contribute toward meeting the urgent need to develop clinical MoDC vaccines for improving Th17 immune competency in listeriosis patients with cancer.

## Conclusion

Listeriosis is a serious infection that causes mortality especially in fetuses and elderly patients with tumors under chemotherapeutic treatment. Examined annual incidences of listeriosis in Gipuzkoa and Cantabria indicated 1.86 and 1.71 cases per 100,000 inhabitants. Considering the reduced Th17 immunocompetency as the main risk factor of listeriosis, biomarkers of poor prognosis, such as high sensitivity to hypervirulent *Listeria* clones, low IL-17A/IL-6 ratios, and anti-GAPDH_1–22_ antibodies should assist in reducing listeriosis incidence. They can also contribute to listeriosis epidemiology and select vaccine antigens and vectors that improve innate and specific immune abilities of patients at high risk of listeriosis.

### Statistics

For all laboratory analyses, *in vitro* and *in vivo* virulence assays, blood tests and antibodies assays *via* ELISA, a Student’s *t*-test was applied. All analyses were performed in triplicate, and results expressed as the mean ± SD calculated. *P* ≤ 0.05 was considered significant. The GraphPad software was used for graph generation. ANOVA was applied for cytokine measurements according to the manufacturer’s instructions.

## Ethics Statement

This study was approved by the Ethical Committee for Clinical Research of Cantabria at Instituto de Investigación Marqués de Valdecilla (Santander, Spain) with the reference number 2014.228 (Acta 20/2014, dated in 2014). All participants signed the Informed Consent documents, and these documents are in the custody of physicians in accordance with the Spanish Law (Ministry of Health). The study was carried out in accordance with the Guide of Care and Use of Laboratory Animals of the Spanish Ministry of Science and Innovation. The Ethical Committee of Animal Experiments of the University of Cantabria approved the protocol (permit number: 2012/06) that followed Spanish legislation (RD 1201/2005). Surgeries were performed under sodium pentobarbital anesthesia, and all efforts were made to minimize suffering.

## Author Contributions

CA-D, planned, directed the study, performed the meetings with all participants to arrange the performance of the study and acted as a corresponding author. ORCID code: 0000-0002-4585-6959. RC-G, performed the study and collected all samples from both Hospitals. HT-N helped with the performance of the study. JM collected the listeriosis samples from patients: bacteria isolates, cells, sera, LCR, blood tests, and clinical data of Hospital Universitario Donostia and performed the Listeria serotyping. CG-R collected the listeriosis samples from patients: cells, sera, LCR, blood tests, and clinical data of Hospital Universitario Marqués de Valdecilla. JC-M collected the Listeria isolates of Hospital Universitario Marqués de Valdecilla and performed the Listeria serotyping. EF-C helped with the cytokine analysis and dendritic cell preparation. MA-G helped to collect the listeriosis samples from patients: bacteria isolates, cells, sera, LCR, blood tests, and clinical data of Hospital Universitario de Donostia and cited the listeriosis patients. MF directed and cited the listeriosis patients, agreement documents from patients and directed the collection of clinical samples and data from listeriosis patients at Hospital Universitario Marqués de Valdecilla. LM-M directed the collection of the *Listeria* isolates from listeriosis patients and the serotyping at Hospital.

## Conflict of Interest Statement

The authors declare that the research was conducted in the absence of any commercial or financial relationships that could be construed as a potential conflict of interest.
