# Supplementary material for: Biomarker Tools to Design Clinical Vaccines Determined from a Study of Annual Listeriosis Incidence in Northern Spain
Source: Front Immunol. 2016 Nov 29;7:541. doi: 10.3389/fimmu.2016.00541 (PMC5126465; doi:10.3389/fimmu.2016.00541)
Supplement: Supplementary file 1 [file Table_1.docx]

**SUPPLEMENTAL FILE.**

**Human listeriosis: epidemiology, immunity and vaccines.**

Clinical manifestations of perinatal listeriosis are mainly meningitis but also jaundice, rashes, respiratory distress and lethargy are detected, being granulomatosis infantiseptica with micro-abscesses and granuloma in livers, spleens and lungs the most severe listeriosis form. Nonperinatal listeriosis presents meningitis, rhombencephalitis, meningoencephalitis, brain abscess or bacteraemia. Listeriosis treatment with ampicillin in combination with gentamicin usually requires long hospitalization periods and fatal outcomes are not prevented. In fact, the high mortality of nonperinatal listeriosis cases appears related to the immune status of the patient and the virulence of the microorganism [34-36]. Hypervirulent *Listeria monocytogenes* strains isolated from clinical patients and especially from outbreaks [5-8,13,14,17,18] have developed a new concept in listeriosis epidemiology, the identification of gene clusters mediating CNS and placental tropisms, referred as complex clones (CC), that combines biodiversity and virulence heterogenicity of clinical isolates [14,17]. Genotyping and virulence assays of clinical isolates would reveal the presence of hypervirulent clones, risk factors to consider in listeriosis.

Another risk factor is the reduced immunocompetence of patients [12]. In this regard, to evaluate the immune status of each listeriosis patient and design prophylactic measures, we should consider the two arms of the immune system, innate and specific responses. Phagocytes as monocytes/macrophages, neutrophils and DC, NK cells, γδ T cells and innate CD8^+^ T cells participate in early innate immune responses. These cells induce specific cytokine patterns that condition the bacterial load. Pro-inflammatory Th1/Th17 cytokines are microbicidal (*i.e.,* TNF-α, IFN-α/β, IFN-γ, IL-1β, IL-12, IL-17A), while anti-inflammatory Th2 cytokines promotes the pathogen dissemination (*i.e.,* IL-4, IL-6 or IL-10) [25,27]. Therefore, the host cytokine pattern might provide predictive information of prognosis in listeriosis. In this regard, high levels of IL-6 or TNF-α are associated with perinatal listeriosis [33,34]. Also IL-17A, a pro-inflammatory cytokine produced by γδ T cells, recruits neutrophils to the inflammation sites and controls this pathogen dissemination [24, 28, 37]. Therefore, ratios of Th1-Th17/Th2 cytokines might be biomarkers of listeriosis severity.

Pathogen-specific CD4^+^ and CD8^+^ T cells generated in listeriosis are essential to clear the infection and confer protection [20,27]. However, pathogen-specific antibodies played only a modest and no protective role [26] but they might serve as markers of pathogen-specific T cell responses and provide epitopes to include in vaccine designs [20,21,30]. *Listeria monocytogenes* intracellular life cycle covers several stages: host adhesion and invasion, vacuole interference and escape, multiplication and motility and intercellular spread. The completion of each stage depends on the activity of specialized bacterial factors, some of them, contributes to evasion of immune defences and appear as good candidates for vaccines [38]. DC vaccines loaded with epitopes of two virulence factors, LLO_91-99_ and LLO_189-201_ epitopes of listeriolysin O (LLO, Lmo0202) and GAPDH_1-22_ epitope of gliceraldehyde-3-phosphate dehydrogenase (GAPDH, Lmo2459) confer listeriosis protection [20,21,39]. Administration of autologous DC as cancer vaccines also function as personalized therapies that present several benefits, lack of rejection, safety, unlimited epitope loading capacity and abilities to modulate Th1/Th2 immune patterns [32]. Therefore, autologous DC vaccines prepared for patients with tumours at high risk of listeriosis presented dual action as prophylactic and therapeutic tools, the latter based in DC abilities to break tumour immunotolerance and promote strong anti-tumour immune responses [22,40, 41].

**Materials and Methods.**

*Bacteria.* EGD (ATCC) (serotype 1/2a) and 10403S (serotype 1/2a) *L. monocytogenes* strains (D.A. Portnoy, Berkley University, CA) were used as standard strains. Invasive *Listeria monocytogenes* from clinical isolates were grown in BHI medium and stored at -80 ºC in BHI-10% glycerol at 1 x 10^10^ CFU/ml.

*Peptides*. *Listeria monocytogenes* GAPDH_1-22_ peptide sequence was obtained from Lmo2459 locus name (MTVKVGINGFGRIGRLAFRRIQ). GAPDH_1-22_ peptide was previuosly described as a relevant epitope of GAPDH virulence factor of this pathogen [29]. *Listeria monocytogenes* LLO_189-201_ peptide sequence was obtained from Lmo0202 locus name (WNEKYAQAYPNVS) and was previously used as binder of MHC-II molecules [21,30,42]. GAPDH_1-22_ peptide was synthesized and purified by *F. Roncal* (CNB. CSIC. Madrid) after HPLC and Mass Spectrometry using a MALDI-TOF Reflex^TM^ IV mass spectrometer (Bruker Daltonics, Bremen, Germany). Peptide purity was >95% after HPLC.

*Microbiological characterization of invasive Listeria monocytogenes.* We collected invasive *L. monocytogenes* from 15 clinical isolates and serotyped the strains by agglutination (Listeria-O-antisera, Difco) and performed multilocus sequence typing (MLST) to establish *L. monocytogenes* sequence types (ST) using the primers and conditions described on the Pasteur Institute web page (http://bigsdb.pasteur.fr/listeria/listeria.html) [43-45]. Briefly, internal fragments of the seven housekeeping genes for *abcZ* (ABC transporter), *bglA* (beta glucosidase), *cat* (catalase), *dapE* (succinyl diaminopimelate desuccinylase), *dat* (D-amino acid aminotransferase), *ldh* (L-lactate dehydrogenase), and *lhkA* (histidine kinase) were amplified using the primers and PCR conditions as described in the *Listeria* MLST web page and sequenced in both directions using the universal sequencing primers oF (GTTTTCCCAGTCACGACGTTGTA) and oR (TTGTGAGCGGATAACAATTTC). Sequences were matched with those included in the *Listeria* MLST database and a number was obtained for each allele. Combination of allelic numbers returned a specific sequence type (ST) number assigned to a particular Clonal Complex (CC).

*Animals.* We used female C57BL/6 mice from our animal facilities at the University of Cantabria at 8–12 weeks old.

*Isolation of MoDC, differentiation and activation.* Monocytes (Mo) from healthy donors or listeriosis patients with tumours were first isolated as leukocytes from a Ficoll gradient of whole blood cells (EDTA-containers). Leukocytes recovered from the interphase were washed twice in Hank´s buffered solution and prepared in MACS^TM^ buffer (PBS-0.5% BSA-2 mM EDTA) with microbeads conjugated to mouse IgG2a monoclonal anti-human CD14 antibody (Miltenyi). CD14^+^ positive cells were selected using MACS^TM^ columns (Miltenyi, Bergisch Gladbach, Germany). FACS analysis following CD14^+^-MACS^TM^ selection indicated 99% of CD45^+^CD14^+^ cells positive cells. MoCD14^+^ cells were differentiated to MoDC at 1 x 10^6^ cells/ml in 6-well plates (Falcon^TM^) during 7 days using GM-CSF (50 ng/ml) and IL-4 (20 ng/ml) in RPMI-20%FCS medium. All differentiated cells were 98% CD45^+^DR^+/-^CD86^-^CD14^-^ positive cells using specific monoclonal antibodies (Miltenyi, Bergisch Gladbach, Germany). These cells were used for *in vitro* virulence analysis (*see below*). MoDC were activated with 50 µg/ml of GAPDH_1-22_ peptide prepared in RPMI-20% FCS medium for 16 h to obtain the following homogeneous cell surface phenotype of activated cells MoDCact, 90% of CD45^+^DR^+^CD86^+^CD14^-^ positive cells. Supernatants of MoDCact were filtered and stored at -80ºC to measure cytokines (*see below*) and cells will be considered vaccine vectors for further assays.

*In vitro virulence of clinical L. monocytogenes isolates.* Human monocyte-derived dendritic cells (MoDC) were obtained from healthy donors, CD14^+^ selected and differentiated with GM-CSF and IL-4 for 7 days as above. MoDC were 99% CD45^+^DR^+^CD11c^+^CD14^-^ positive cells [19]. MoDC were infected at a multiplicity of infection, MOI, of 20:1 (bacteria: cells). *In vitro* virulence was calculated as replication index (RI) representing the ratio of CFU at 16 h post-infection to CFU at 1 h post-infection [20-22].

*In vivo virulence of clinical L. monocytogenes isolates.* C57BL/6 female mice were inoculated intravenously (*i.v)* with 100 µL of a *L. monocytogenes* bacterial suspension of each clinical isolate in saline (5 × 10^3^ CFU/mice). 72 hours post-inoculation mice were sacrificed and spleens recovered, homogenized and viable (CFU) bacteria examined in blood agar plates. Results are expressed as the mean of CFU ± SD. All data were performed in triplicate and we performed three independent experiments.

*FACS analysis.* Cell surface markers of MoDC were analyzed by FACS using the following antibodies: anti-DR-FITC, anti-CD45-PerCP, anti-CD86-brilliant blue and anti-CD14-PE (Miltenyi, Bergisch Gladbach, Germany). Cytokines in patients or donors sera and MoDC supernatants were quantified using the CBA kit (BD Biosciences, San Jose, CA, USA). IL-17A/IL-6 ratios are expressed as the mean of ratio units (U) of triplicates ± SD. ANOVA was applied to these samples according to manufacture´s instructions. Data were analysed using the FlowJo software.

*ELISA to measure GAPDH_1-22_ titers.* GAPDH_1-22_ or LLO_189-201_ peptides (50 µg/ml) were coated to 96-well plates in carbonate buffer (pH 8.0) overnight at 4ºC. Plates were washed and incubated with 1 mg/ml of BSA (fraction V) to saturate all sites in the plates. Sera of listeriosis patients were 1/10 diluted and GAPDH_1-22_ coated plates incubated with diluted sera for 2 hours at RT as described [21, 30]. Reactions were developed with goat anti-human IgG and absorbance analysed at 450 nm. Results are presented as optical units (OD) and mean values ± SD of triplicate experiments.

**References.**

34.- Vazquez-Boland JA, Kuhn M, Berche P, Chakraborty T, Dominguez-Bernal G, Goebel W, Gonzalez-Zorn B et al. Listeria pathogenesis and molecular virulence determinants Clin. Microbiol Rev. 2001; 14(3): 584-640.

35.- Ramana KV, Mohanty SK. Human listeriosis: an update. Am J Epidemiol Infect Dis. 2013; 1(4): 63-66.

36.- Drevets DA, Bronze MS. Listeria monocytogenes: epidemiology, human disease and mechanisms of brain invasion. FEMS Immunol Med Microbiol. 2008; 53: 151-165.

37.- Jim W, Dong C. IL-17 cytokines in immunity and inflammation. Emerg Microbes Infect. 2013; 2, e60. doi: 10.1038/emi.2013.58.

38.- Camejo A, Carvalho F, Reis O, Leitao E, Sousa S, Cabanes D. The arsenal of virulence factors deployed by Listeria monocytogenes to promote its cell infection cycle. Virulence. 2016; 2:5, 379-394.

39.- Calderon-Gonzalez R, Frande-Cabanes E, Tobes R, Pareja E, Alaez-Alvarez L, Alvarez-Dominguez C. A dendritic cell targetted vaccine loaded with a glyceraldehyde-3-phosphate-dehydrogenase peptide proposed for individuals at high risk of listeriosis. J Vaccines Vaccin. 2015; 6:266. doi: 10.4172/2157-7560.1000266.

40.- Calderon-Gonzalez R, Bronchalo-Vicente L, Freire J, Frande-Cabanes E, Alaez-Alvarez L, Gomez-Roman J, Yañez-Diaz S, Alvarez-Dominguez C. Exceptional anti-neoplastic activity of a dendritic-cell-targetted vaccine loaded with a Listeria peptide proposed against metastatic melanoma. Oncotarget. 2016; 7,13: 16855-65.

41.- Mitchell DA, Batich KA, Gunn MD, Huang MN, Sanchez- Perez L, Nair SK, Congdon KL, Reap EA, Archer GE, Desjardins A, Friedman AH, Friedman HS, Herndon JE 2nd, et al. Tetanous toxoid and CCL3 improve dendritic cell vaccines in mice and gliobastoma patients. Nature. 2015; 519:366–9. doi:10.1038/nature14320.

42.- Rodriguez-Del Rio E, Frande-Cabanes E, Tobes R, Pareja E, Lecea-Cuello MJ, Ruiz-Saez M, Carrasco-Marin E, Alvarez-Dominguez C. The intact structural form of LLO in endosomes cannot protect against listeriosis. Int J Biochem Mol Biol. 2011; 2(3): 207-218.

43.- Doumith M, Buchrieser C, Glaser P, Jacquet C, Martin P. Differentiation of the major *Listeria monocytogenes* serovars by multiplex PCR. J Clin Microbiol. 2004; 42: 3819-22.

44.- Cantinelli T, Chenal-Francisque V, Diancourt L, Frezal L, Leclercq A, Wirth T, Lecuit M, Brisse S. ["Epidemic clones" of Listeria monocytogenes are widespread and ancient clonal groups.](https://www.ncbi.nlm.nih.gov/pubmed/24006010) J Clin Microbiol. 2013; 51(11): 3770-9. doi: 10.1128/JCM.01874-13.

45.- Stessl B, Rückler I and Wagner M. Multilocus sequence typing (MLST) of Listeria monocytogenes. Methods Mol Biol. 2014;1157:73-83. doi: 10.1007/978-1-4939-0703-8_6.

**Table S1.- Immune biomarkers in listeriosis patients.**

| **Patients code^a^-Age^b^. Period 2014-2015** | **Cytokines^c^ and antibodies^d^ in sera** | | |
| --- | --- | --- | --- |
|  | **IL-17A/IL-6** | **IL-10** | **anti-GAPDH_1-22_** |
| *HUD005-74 (*died*) | **1.20±0.1 | 4.9 ± 0.2 | 0.02 ± 0.1 |
| *HUD006-60 | **1.20±0.2 | 5.1 ± 0.3 | **0.38 ± 0.2 |
| *HUD013-59 | 1.6 ± 0.2 | 5.3 ± 0.2 | **0.31 ± 0.1 |
| *HUMV006-76 (*died*) | **1.75±0.1 | 4.1 ± 0.2 | 0.05 ± 0.1 |
| *HUMV007-51 | *2.80±0.1 | 1.6 ± 0.1 | *0.5 ± 0.2 |
| *HUMV009-54 | 2.80±0.2 | 4.6 ± 0.1 | **0.7 ± 0.2 |
| *HUMV013-59 | **1.20±0.1 | 3.8 ±0.1 | **0.2 ± 0.1 |
| HUD012-65 (*Autoimmune*) | 22 ± 0.4 | 2.49 ± 0.1 | 1.9 ± 0.2 |
| HUMV012-84 (*Autoimmune*) | 19 ± 0.2 | 2.45 ± 0.1 | 4.5 ± 0.2 |
| HUD009-74 (*Elderly*) | 9.2 ± 0.2 | 2.89 ± 0.1 | 1.8± 0.1 |
| HUD010-90 (*Elderly*) | 8.1 ± 0.5 | 2.91 ± 0.2 | 2.3 ± 0.2 |
| HUD007-30 (*Miscarriage*) | 7.4 ± 0.6 | 2.80 ± 0.1 | 1.5 ± 0.2 |
| HUMV010-57 (*Kidney transplant*) | 28 ± 0.1 | 2.40 ± 0.2 | 3.6 ± 0.1 |
| HUD011-54 (*None*) | 9.0 ± 0.3 | 2.48 ± 0.1 | 2.6 ± 0.1 |
| **CONTROL-52** | **4.83 ± 0.2** | **2.40 ± 0.1** | **0.16 ± 0.1** |
| **Patients code-Age. Selection in 2012-14^e^** | **Cytokines and antibodies in sera** | | |
|  | **IL-17A/IL-6** | **IL-10** | **anti-GAPDH_1-22_** |
| *HUD001-57 | **3.25±0.1 | 4.0 ± 0.1 | **1.14 ± 0.1 |
| *HUMV002-65 | 0.95±0.1 | 2.4 ± 0.1 | *0.80 ± 0.2 |
| *HUMV005-49 | **1.59±0.2 | 4.0 ± 0.1 | **0.50 ± 0.1 |
| HUD002-36 (*Autoimmune*) | 141 ± 0.5 | 2.8 ± 0.2 | 2.3 ± 0.1 |
| HUMV001-89 (*Autoimmune*) | 10.2 ± 0.2 | 3.1 ± 0.1 | 2.5 ± 0.3 |
| HUMV003-60 (*Autoimmune*) | 22.9 ± 0.2 | 2.9 ± 0.2 | 3.0 ± 0.4 |
| HUD003-30 (*Miscarriage*) | 11 ± 0.1 | 2.1 ± 0.2 | 2.2 ± 0.2 |
| HUD004-32 (*Miscarriage*) | 10.5± 0.1 | 2.1 ± 0.1 | 2.0 ± 0.1 |
| HUMV004-56 (*Hepatic transplant*) | 23.5±0.5 | 3.1 ± 0.2 | 3.0 ± 0.5 |
| **CONTROL-51** | **4.83±0.2** | **2.30 ± 0.1** | **0.15 ± 0.1** |

^a^Listeriosis patients in the period of August 2014-September 2015 and highlighted with asterisks tumour patients. Patients are identified by internal codes. HUD: Hospital Universitario de Donostia (San Sebastian, Gipuzcoa), HUMV: Hospital Universitario Marqués de Valdecilla (Santander, Cantabria). ^b^Age of the patient in years. Control parameters correspond to healthy donors of 51 or 52 years of age. ^c^Cytokine concentration in sera (pg/ml) measured by flow cytometry. Ratios of IL-17A/IL-6 are expressed as the mean of ratio units (U) of triplicates ± SD. **, *P* ≤ 0.01; *, *P* ≤ 0.05. ^d^Sera of listeriosis patients were examined for peptide-ELISA (anti-GAPDH_1-22_) as described in *methods* [21, 30]. Results are optical units (OD) and the mean of triplicates ± SD. **, *P* ≤ 0.01; *, *P* ≤ 0.05. ^e^Listeriosis patients in the selection 2012-14. Parenthesis indicates the clinical manifestations.
